# Potential role of CTHRC1 in renal fibrosis

**DOI:** 10.1007/s11033-026-12886-5

**Published:** 2026-10-16

**Authors:** Gulzhan Bolebay, Raushan Ayekenova, Roza Izimova, Balzat Sarimbayeva, Galym A. Shegenov, Assem Kaliyeva Mukhtarkhanovna, Arman Issimov, Askhat Myngbay, Nikki A. Copeland, Makhabbat Amanbayeva, John G. Hardy

**Affiliations:** 1 Department of Biology, K. Zhubanov Aktobe Regional University, Aktobe, Kazakhstan; 2 “City Multidisciplinary Hospital, No. 2”, Astana, Kazakhstan; 3 Astana Medical University, Astana, Kazakhstan; 4 Asfendiyarov Kazakh National Medical University, Almaty, Kazakhstan; 5 Department of Biology, Abai Kazakh National Pedagogical University, Almaty, Kazakhstan; 6 Department of Biomedical Sciences, Lancaster University, Lancaster, UK; 7 Department of Chemistry, Lancaster University, Lancaster, UK

**Keywords:** CTHRC1, Renal fibrosis, Fibroblasts, Tubular epithelial cells

## Abstract

Renal fibrosis is a major morphological feature of chronic kidney disease and is considered an important pathogenetic process that leads to a decline in renal function in the progressive course of the disease. It is characterized by excessive accumulation of extracellular matrix, tubular atrophy, interstitial inflammation, capillary thinning, and nephron structural disruption. The development of fibrosis involves transforming growth factor-β (TGF-β), Wnt/β-catenin, inflammatory mediators, and intercellular communication between macrophages and activated fibroblasts. TGF-β, as one of the main driving factors of renal fibrosis, enhancing the activation of myofibroblasts and the accumulation of extracellular matrix proteins such as collagen and fibronectin through Smad-dependent and Smad-independent pathways. Collagen Triple Helix Repeat Containing 1 (CTHRC1), a secreted glycoprotein, was initially described in vascular remodeling and tissue repair processes after injury, and later studies began to consider it as a regulatory molecule in fibrotic diseases. The high expression of CTHRC1 protein in renal tissue in tubular cells and undetectable levels in glomerular cells provide a basis for investigating its association with tubulointerstitial injury in the kidney. CTHRC1 may be involved in fibroblast migration, tissue remodeling, extracellular matrix metabolism, and interaction with the TGF-β/Wnt signaling systems during kidney fibrogenesis. This review discusses the potential role of CTHRC1 in renal fibrosis, its relationship with the TGF-β and Wnt/β-catenin pathways, and its potential as a diagnostic biomarker and therapeutic target in chronic kidney diseases.

## Introduction

Collagen triple helix repeat containing 1 (CTHRC1) has been identified as an onc-associated protein. Its overexpression correlates with tumor transformation, proliferation, invasive growth, and metastasis in a wide range of human cancers. It has been established that CTHRC1 expression is regulated by many molecular factors, including microRNAs, long non-coding RNAs (lncRNAs), as well as signaling and glycosylating proteins, in particular WAIF1 and DPAGT1 [1]. CTHRC1 is characterized by multilevel and complex interactions with intracellular components and the extracellular matrix implemented through various mechanisms and secretion pathways [1]. CTHRC1 promoted wound healing through recruitment of macrophages and regulation of TGF-β signaling pathway [2]. CTHRC1 also played an important role in bone formation and remodeling [3]. During the healing process of skin lesions, increased regulation of CTHRC1 has been shown to accelerate fibroblast migration to the wound bed and promote the formation of granulation tissue [4]. Chronic kidney disease is characterized by a variety of etiological factors, and understanding its complex pathophysiology is inherently a significant scientific and clinical challenge [5].

One of the chronic kidney diseases is kidney fibrosis. Renal fibrosis is characterized by several pathological processes, including the infiltration of inflammatory cells, the activation and proliferation of fibroblasts, as well as the accumulation of extracellular matrix (collagen, fibronectin, proteoglycans), followed by the replacement of normal fibrous tissue. This leads to impaired tubule function, loss of peritubular capillaries, and restructuring of the renal tissue, which eventually results in renal failure [6]. The level of CTHRC1 in blood plasma was shown to be able to distinguish patients with chronic kidney disease from healthy individuals. The existing research indicates that CTHRC1 plasma levels could be a valuable adjunct biomarker in the diagnosis of CKD [7].

Renal fibrosis is one of the common end-stage pathological processes of chronic kidney disease (CKD) and leads to irreversible decline in renal function. Currently, there are no specifically approved antifibrotic drugs that directly reverse or reverse renal fibrosis. Therefore, treatment strategies are mainly aimed at slowing the progression of fibrosis. Commonly used methods include drugs that inhibit the renin-angiotensin-aldosterone system (RAAS) (angiotensin-converting enzyme inhibitors and angiotensin receptor blockers), sodium-glucose cotransporter-2 (SGLT2) inhibitors, mineralocorticoid receptor antagonists, control of arterial hypertension and diabetes, and lifestyle modification. These approaches slow the decline in renal function and indirectly inhibit fibrotic processes.

The available data indicate that the interaction between CTHRC1 and the TGF-β/Smad signaling pathway is not unidirectional, but rather tissue- and cell-type-dependent. In vascular smooth muscle cells, activation of the TGF-β/Smad pathway increases CTHRC1 expression, while increased CTHRC1 levels, in turn, reduce Smad2/3 phosphorylation and collagen synthesis, forming a possible negative feedback mechanism. A similar antifibrotic effect has also been described in experimental pulmonary fibrosis: CTHRC1deficiency was accompanied by increased TGF-β/Smad activity and excessive collagen deposition. In contrast, in hepatic stellate cells, autocrine CTHRC1 was shown to enhance their activation and promote fibrosis. Taken together, these results suggest that the profibrotic or antifibrotic effects of CTHRC1 depend on the cellular and tissue environment. Therefore, direct extrapolation of mechanisms identified in other organisms to renal fibrosis is not justified until confirmed by kidney-specific functional studies (Table 1).

**Table 1:** List of main studies reflecting potential role of CTHRC1 in different organs and diseases

Tissue / disease	CTHRC1-expressing fibroblast population	Effect	Reference
Liver fibrosis	Activated hepatic stellate cells / myofibroblasts	Pro-fibrotic Activated HSCs → ↑ CTHRC1 → TGF-β signaling → ↑ HSC activation → ↑ fibrosis Anti-fibrotic CTHRC1 knockdown/antibody inhibition → ↓ CTHRC1 activity → ↓ HSC activation → ↓ fibrosis	[8]
Pulmonary fibrosis / IPF / systemic sclerosis lung	CTHRC1⁺ pathological fibroblasts	Pro-fibrotic Lung fibrosis → emergence of CTHRC1⁺ fibroblasts → ↑ collagen expression → accumulation in fibroblastic foci → fibrosis progression	[9]
Cardiac fibrosis / myocardial infarction	CTHRC1⁺ reparative cardiac fibroblasts	Pro-fibrotic/reparative Cardiac injury → CTHRC1-high fibroblasts → profibrotic program → scar localization → cardiac healing/remodeling	[10]
Systemic sclerosis / dermal fibrosis	Human dermal fibroblasts	Anti-fibrotic Recombinant CTHRC1 → ↓ TGF-β1-induced collagen production in dermal fibroblasts → ↓ bleomycin-induced skin fibrosis	[11]
Keloid / skin fibrosis	CTHRC1⁺ fibroblasts	Pro-fibrotic Macrophages → SPP1/osteopontin → activation of CTHRC1⁺ fibroblasts → ↑ collagen production → keloid fibrosis	[12]
Heart + liver fibrosis	CTHRC1-high myofibroblasts	Pro-fibrotic Drebrin-dependent actin organization → MRTF–SRF signaling → ↑ Cthrc1 expression in myofibroblasts → cardiac and hepatic fibrosis	[13]
Crohn’s disease / intestinal fibrosis	CTHRC1⁺ fibroblasts derived from creeping fat	Pro-fibrotic Creeping fat → mechanosensitive CTHRC1⁺ fibroblasts → profibrotic activation → intestinal stricture/fibrosis	[14]
Human IPF	CTHRC1⁺ fibroblasts	Pro-fibrotic Alveolar/inflammatory fibroblasts → CTHRC1⁺ fibroblast state → ↑ ECM biosynthesis and cell–cell signaling → accumulation in fibroblastic foci → lung fibrosis	[15]

### Molecular structure and regulation of CTHRC1

The *CTHRC1* gene is located on human chromosome 8q22.3 and encodes several isoforms that differ in molecular weight and intracellular location. Secreted forms of CTHRC1 undergo posttranslational glycosylation before being secreted from the cell, resulting in a higher molecular weight than their intracellular counterparts. The structural specificity of the CTHRC1 protein is associated with its ability to form complex molecular complexes. It can self-assemble into higher-order structures, organizing itself into dimers or trimers. This property may contribute to the stability of CTHRC1 in the extracellular environment, its biological activity, and its interaction with various signaling pathways [16, 17].

The protein has several functional regions: an N-terminal region necessary for its secretion; a collagen-like domain with characteristic Gly-X-Y repeats, which plays a role in oligomerization; and a highly conserved COOH-terminal domain [18]. The initial, N-terminal section of the protein contains a hydrophobic signaling peptide (about 20–30 amino acids), which directs the synthesized polypeptide chain into the secretory apparatus of the cell, ensuring its subsequent presence outside the cell. The structural organization of the central domain of the protein, represented by collagen-like Gly-X-Y triplets, plays a key role in its oligomerization (formation of dimers and trimers). These repeats not only contribute to the assembly of the molecule, but also increase its affinity for the components of the extracellular matrix. This structural feature confirms the functional significance of CTHRC1 in the processes of cellular locomotion and structural restructuring (remodeling) of tissues. The final C-terminal domain is characterized by a high degree of conservation in multicellular organisms. Although its primary amino acid sequence does not show significant similarity to other proteins, structural modeling suggests that it has folds similar to the domains of the C1q family and the non-collagen (NC) domains of collagens, which are involved in the construction of extracellular networks [18].

### CTHRC1 and fibrotic signaling pathways

#### Interaction with TGF-β/Smad signaling

Fibrotic pathologies of humans are an urgent and large-scale medical and biological problem of global importance. Their high prevalence and significant impact on morbidity and mortality rates make them the subject of close attention [19]. However, despite active scientific research, the pathogenesis of fibrosis remains poorly understood. This is due to the wide variability of the causes and clinical manifestations of fibrotic conditions. The lack of specific and proven biomarkers also poses serious obstacles to early diagnosis and monitoring of disease progression. Moreover, there are currently no effective therapeutic strategies that can affect the course of fibrotic diseases [20]. Thus, there is an urgent need for further in-depth investigation of the molecular mechanisms of fibrosis and the development of new treatment methods. A wide range of molecular mechanisms and changes in the tissue microenvironment are involved in the formation of fibrosis. In particular, transforming growth factor β (TGF-β), platelet-derived growth factor (PDGF), connective tissue growth factor (CTGF), interleukin-4 (IL-4), interleukin-13 (IL-13) and other cytokines have pronounced chemotactic and proliferative effects on fibroblasts, as well as regulate synthesis and degradation of extracellular matrix components, primarily collagen [21]. The imbalance between the processes of collagen formation and destruction, in turn, is closely related to the initiation and progression of the fibrous process.

Among the listed factors involved in the development of fibrosis, transforming growth factor β (TGF-β) plays a key pathogenetic role [21]. With persistent inflammation, in conditions of chronic tissue damage, a stable hyperactivation of the TGF-β signaling cascade occurs. Activated TGF-β not only mediates the canonical SMAD signaling pathway, but also induces activation of the non–canonical PI3K-AKT-mTOR pathway regulating transcription processes [22]. Activation of these signaling cascades, in turn, promotes the induction of epithelial-mesenchymal transition (EMT) and increased accumulation of extracellular matrix components, which is a characteristic feature of progressive fibrosis [23]. The fibrogenic process is largely regulated by transforming growth factor β1 (TGF-β1), while it has been shown that a specific blockade of the signaling pathway TGF-β1/Smad3 has a pronounced therapeutic potential and is able to effectively suppress the development of fibrosis in various tissues [24]. TGF-β1 is a secreted homodimeric protein that regulates a wide range of cellular processes, including proliferation, differentiation, migration, and apoptosis, and initiates the canonical signaling cascade TßRII-ALK5-Smad2/3 in most cell types [25].

**Fig. 1:**
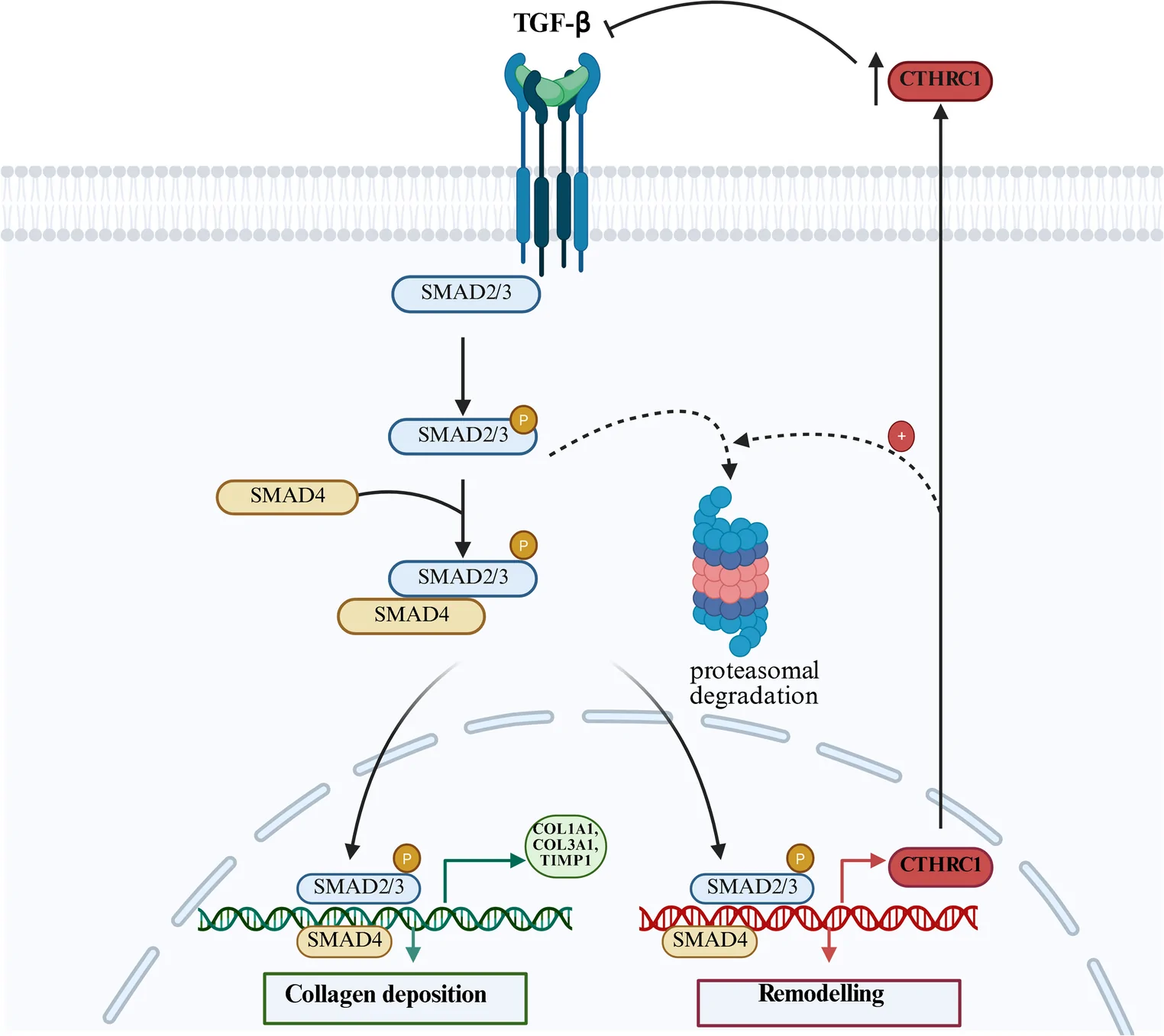
Postulated TGF-β/Smad signaling of Proximal tubular epithelial cells (PTEC). This figure illustrates that injured PTEC activates TGF-β/Smad signaling which leads to secretion of CTHRC1. Elevated concentration of CTHRC1 in tubulointerstial area eventually initiates WNT signalling pathway on fibroblasts transforming them to myofibroblasts. CTHRC1 was shown as a regulator of tissue fibrosis through enhancement or inhibition of transforming growth factor-β (TGF-β)/Smad signaling and extensive crosstalk with multiple profibrotic signaling pathways. * Created in BioRender. Myngbay, A. (2026) https://BioRender.com/xsqlxg9

CTHRC1 is considered as a factor capable of inhibiting TGF-β signaling in a cell-specific manner. It has been proposed that this effect is one of the key mechanisms by which CTHRC1 suppresses the expression of types I and III collagen [26]. The antifibrotic properties of CTHRC1 are of considerable therapeutic interest since fibrosis of various organs remains a serious and still unresolved clinical problem. Studies in transgenic mouse models have shown that enhanced phosphorylation of Smad2/3 in the downstream signaling cascade of TGF-β leads to the induction of mRNA and CTHRC1 protein expression [18]. In addition, overexpression of CTHRC1 significantly suppresses the signal transduction of TGF-β by reducing the level of phosphorylated Smad2/3 [26], indicating the presence of a regulatory feedback between CTHRC1 and the TGF-β signaling pathway (Fig. 1).

Activation of the Wnt signaling pathway is considered as one of the fundamental molecular mechanisms that determine the induction of osteoblastic activity and bone remodeling [3, 27]. The components of the Wnt signaling pathway are critically necessary for embryonic development and continue to play a key role in the processes of regeneration and renewal of adult tissues, including the lymphoid system, colon epithelium, skin, hair follicles, and bone tissue [28]. The accumulated data indicates that during the embryonic development of the kidneys, the Wnt ligands Wnt9b and Wnt4 are key initiators of the Wnt signaling pathway. Their activation causes a series of intracellular signals necessary for the transition of nephrogenic mesenchymal cells into epithelial cells, which subsequently form nephrons [29]. In mammals, Wnt signaling is primarily carried out within the framework of local cellular interaction, that is, between cells in close proximity, rather than through long-range remote mechanisms [30].

**Fig. 2:**
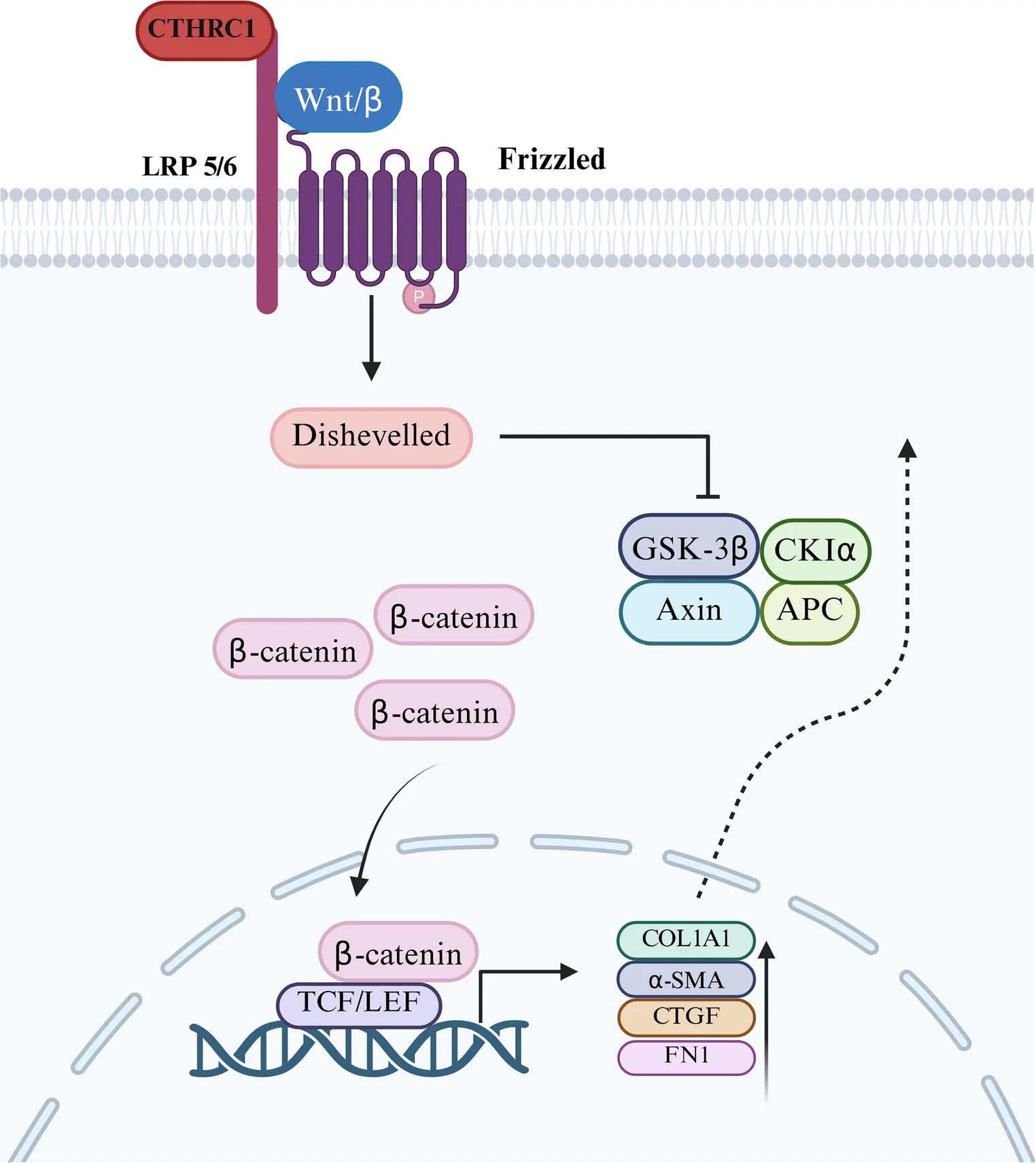
Modulation of Wnt signalling on activated fibroblasts and myofibroblasts. This figure illustrates the molecular mechanisms through which CTHRC1 enhances canonical Wnt/β-catenin signaling, resulting in activation of profibrotic gene expression, extracellular matrix (ECM) accumulation, and progressive tissue fibrosis. CTHRC1 functions as a secreted matricellular protein that amplifies multiple steps of the Wnt signaling cascade, including ligand stabilization, receptor activation, inhibition of the β-catenin destruction complex, and transcriptional activation of Wnt target genes. * Created in BioRender. Myngbay, A. (2026) https://BioRender.com/3unl54b

The main role of CTHRC1 is as an important ligand in the assembly of complex molecular structures consisting of the proteins Wnt, Fzd and Ror2. In addition, it promotes the launch of the PCP signaling pathway, which plays a key role in the development and organization of tissues. The coculture IP method was used to analyze the interactions of CTHRC1 with Wnt signaling components (Wnt, Fzd, LRP, and Ror2), which showed that CTHRC1 binds directly to Wnt and Ror2, enhancing activation of the PCP pathway and simultaneously suppressing the canonical Wnt/β-catenin pathway (Fig. 2) [31]. In addition, it was found that the functional activity of CTHRC1 is due to a highly conserved C-terminal domain with a length of about 200 amino acids [32].

### Fibrosis in other organs

Along with the well-studied involvement of CTHRC1 in the pathogenesis of cardiovascular diseases, malignancies, and rheumatoid arthritis, research increasingly links CTHRC1 protein to the development of fibrous processes in systemic lupus erythematosus, muscular dystrophies, and fibrosis of various organs, including the lungs, heart, liver, and skin. Currently, special attention is being paid to the role of CTHRC1-positive fibroblasts in pulmonary fibrosis. Single-cell transcriptomic studies and cell line tracing methods have shown that a subpopulation of pulmonary fibroblasts produce a pathological extracellular matrix that underlies fibrogenesis [33]. Idiopathic pulmonary fibrosis is characterized by excessive collagen deposition and progressive decrease in lung function, which is associated with aberrant activation of the TGF-β and Wnt signaling pathways and subsequent activation of Smad2/3 [34]. The CTHRC1 protein inhibits activation of Smad2/3 and may limit the development of fibrosis. In an experiment on mice with knockout of the *Cthrc1* gene and wild-type animals, fibrosis was induced by intratracheal administration of bleomycin. The Cthrc1⁻/⁻ mice showed higher levels of TGF-β and hydroxyproline, as well as a marked decrease in lung compliance compared with the control group [35]. In addition, beta-catenin activation was observed in these animals in tissue remodeling zones, which indicates the protective role of CTHRC1 in limiting collagen formation and reducing the severity of fibrosis [35].

Using bleomycin induced lung damage revealed that a lack of CTHRC1 leads to a persistent increase in the concentration of TGF-β in bronchoalveolar lavage fluid, and this effect is observed regardless of the nature of exposure. During the experiment, it was found that an increase in TGF-β is associated with increased collagen deposition and the development of pulmonary fibrosis. Since the initial level of TGF-β in *CTHRC1⁻/⁻* animals was already elevated, the administration of bleomycin did not lead to a significant increase in TGF-β., as a result, the increase in fibrosis is less pronounced, which was reflected in a lower increase in hydroxyproline. In addition, an increase in the number of pSmad2⁻positive cells was detected in the lungs of *CTHRC1⁻/⁻* animals 14 days after exposure, indicating activation of the Smad-dependent signaling pathway [35]. Based on the data obtained, conclusions can be drawn about the regulatory role of CTHRC1in TGF-β-mediated pulmonary fibrosis and it is considered as a potential therapeutic target.

CTHRC1 is expressed predominantly in pathological lung fibroblasts and is almost absent in healthy tissues. Patients with IPF exhibited notably elevated serum CTHRC1 levels compared to the control group, which correlated with the disease’s severity. According to the CTHRC1 + single-cell RNA sequence, fibroblasts had high expression of extracellular matrix proteins and are localized in fibroblast foci, supporting their profibrotic phenotype. The results of dynamic monitoring and comparative analyses with tissue expression levels suggest that the amount of CTHRC1 in the bloodstream may reflect the level of activity and accumulation of pathological fibroblasts in lung tissue. These data indicate that CTHRC1 may be a promising prognostic biomarker in idiopathic pulmonary fibrosis. In particular, it may allow assessing the severity of the disease, determining the activity of the fibrotic process, and clarifying the clinical prognosis of patients [36].

Single-cell transcriptome analysis identified a unique population of cardiac fibroblasts that are involved in ventricular remodeling during cardiac fibrosis. This cell group has a unique transcriptome profile characterized by high expression of the *CTHRC1* gene. The high levels of CTHRC1 indicate that these fibroblasts play an important role in the formation of fibrotic scar tissue and the accumulation of extracellular matrix. Thus, these fibroblasts can be considered as one of the key cellular components contributing to the development of cardiac fibrosis and pathological changes in myocardial structure. The nonclassical TGF-β pathway is cardiac fibrosis, and knockout of CTHRC1 reduces the effectiveness of cardiac recovery. The preservation of this CTHRC1 + subgroup has been confirmed in a preclinical pig model and in patients, which underlines its clinical significance [37].

Similarly, liver stellate cells (HSC), representing a nonparenchymatous population of liver cells, are considered the main source of extracellular matrix, and the TGF-β1/Smad3 signaling pathway acts as a key pro-fibrogenic factor that triggers and supports the process of fibrogenesis [38]. CTHRC1 promotes the activation, migration and contractility of liver stellate cells (HSCs) through TGF-β and the non-canonical Wnt signaling pathway [39]. Unlike direct blocking of TGF-β, targeting CTHRC1 makes it possible to suppress HSCs activation and fibrosis without undesirable side effects [40].

### Clinical trials on CTHRC1

Currently, there are very few registered clinical trials evaluating CTHRC1 as a direct therapeutic target in kidney diseases, including chronic kidney disease, diabetic kidney disease, or renal fibrosis. A search of the databases ClinicalTrials.gov, WHO ICTRP, EU Clinical Trials/CTIS, and Cochrane Kidney and Transplant indicates that no active interventional trials targeting CTHRC1 have yet been registered in the context of renal fibrosis or CKD/DKD. This suggests that the clinical translation of CTHRC1 in nephrology is still in its early stages and itsvalidation is required in large, prospective biomarker cohorts.

In our previous study comparing CKD patients with healthy controls, plasma CTHRC1 levels were significantly higher in the CKD group and correlated with renal function markers such as 24-hour proteinuria, creatinine, urea, uric acid, and eGFR [7]. Computational studies provide additional evidence to support the potential role of CTHRC1 in renal pathology. Specifically, for renal cell carcinoma, analyses based on open databases such as TCGA, TIMER, Oncomine, UALCAN, Kaplan–Meier Plotter, and TISIDB have shown that CTHRC1 expression is elevated in kidney renal clear cell carcinoma (KIRC) and kidney renal papillary cell carcinoma (KIRP) tissues [41], and is associated with tumor stage, lymph node metastasis, poor prognosis, and immune cell infiltration [41]. This suggests that CTHRC1 may be a prognostic and immune microenvironment-related biomarker in renal cancer, but these findings cannot be directly extrapolated to renal fibrosis because the pathogenesis of cancer and fibrosis is not the same. Additional bioinformatics studies using GEO transcriptome data, STRING, Cytoscape/Gephi, and survival analysis methods identified CTHRC1 as one of the differentially expressed genes associated with metastasis in clear cell renal cell carcinoma; high CTHRC1 expression was associated with poor overall survival in ccRCC patients [42]. Such studies demonstrate the association of CTHRC1 with extracellular matrix, focal adhesion, PI3K–Akt signaling pathway, and immune infiltration, and therefore it may be considered as a candidate molecule that requires further investigation in renal cancer biology. However, in silico studies specifically investigating CTHRC1 in renal fibrosis and CKD are still lacking. However, single-cell RNA-seq, ATAC-seq, and spatial transcriptomics data allow for the high-resolution characterization of matrix-forming fibroblasts and pericytes in renal fibrosis, as well as the fibrotic microenvironment [43]. For example, single-cell studies that have characterized the origin of scar-forming myofibroblasts in human renal fibrosis and initiatives such as the Kidney Precision Medicine Project, which aims to create molecular atlases for CKD/AKI, will allow for the future evaluation of CTHRC1 as a cellular-spatial biomarker of renal fibrosis [44].

In this regard, the next steps are to prospectively assess CTHRC1 levels in patients with CKD, DKD, and biopsy-proven renal fibrosis and compare this with eGFR, albuminuria, histological interstitial fibrosis/tubular atrophy, and spatial transcriptomics data. It is also necessary to analyze the expression of CTHRC1 in fibroblasts, pericytes, tubular epithelium, and immune cells at the single-cell level. Such studies will clarify whether CTHRC1 is simply a marker accompanying the disease or a therapeutic target that functionally contributes to the development of fibrosis. The current literature contains human data on CTHRC1 in renal diseases, but these are mostly limited to observational and model-based studies. Proteomic analysis of mesothelial exosomes from children with FSGS on peritoneal dialysis identified CTHRC1 as a component of a protein profile associated with fibrosis and EMT [45]. Increased CTHRC1 expression and association with clinical and prognostic indicators have been demonstrated in human tissues in renal tumors, including RCC and Wilms tumor [46]. However, human interventional in vivo studies evaluating CTHRC1 as a direct therapeutic target in renal fibrosis or CKD are lacking.

There are also studies in human tissues for renal tumors. For example, in the RCC study, CTHRC1 was shown to be upregulated in primary renal cell carcinoma tissues and RCC cell lines [47]. The human part included qRT-PCR and Western blot analysis of 7 RCC tissues and 7 non-tumorous normal tissues, while the functional evidence is mainly from cellular knockdown models. Therefore, this is an ex vivo/translational study based on human tissue, but not a functional in vivo test in living humans.

There are also human data for CTHRC1 in Wilms tumor: 42 Wilms tumor tissues and 42 adjacent non-tumorous tissues were examined by immunohistochemistry, and 8 patient samples were examined by Western blot and qRT-PCR. High expression of CTHRC1 was associated with tumor size, clinical stage, histopathological type, vascular invasion/metastasis, and shorter survival [46]. This is considered a clinical-prognostic study for kidney cancer, but not direct evidence for the pathogenesis of renal fibrosis or CKD.

### CTHRC1, kidney organoids, and in silico studies

Currently, there is limited dedicated work investigating the role of CTHRC1 in kidney diseases. However, kidney organoids derived from human iPSCs are being actively used to model kidney fibrosis. For example, in kidney organoid models induced by TGF-β1, differentiation of resident fibroblasts into myofibroblasts, upregulation of fibrosis-related genes, and changes in chromatin accessibility have been described using single-cell multiomics [48]. In this model, the SMAD3–EZH2 axis has been shown to be involved in the fibrotic response. In other organoid studies, activation of TGF-β1 signaling in kidney organoids has been shown to induce phenotypes similar to human renal fibrosis, such as tubular atrophy, glomerulosclerosis, and interstitial fibrosis [48]. RNA-seq analysis has revealed alterations in epithelial-mesenchymal transition, inflammation, metabolism, and JAK/STAT signaling pathways, suggesting potential mediators of fibrosis, such as PIM1. Inhibition of PIM1 attenuated fibrotic changes in organoids [48]. Furthermore, iPSC-derived kidney organoids that mimic clinically relevant injury conditions with hypoxia and IL-1β exhibited increased FN1 and collagen type I expression, as well as signs of tubular regeneration [49]. Furthermore, the regulation of p-SMAD3, TAZ/TEAD, and COL1A1 in TGF-β1-induced fibrotic organoid models was investigated, and the bile acid receptor agonist INT-767 was shown to attenuate the fibrotic response [50]. However, most of these organoid studies have not specifically analyzed CTHRC1 as a key molecule. Therefore, the study of CTHRC1 in the organoid context of kidney fibrosis remains an open question. In the future, it will be important to reanalyze bulk RNA-seq and single-cell RNA-seq data from renal organoids exposed to TGF-β1, hypoxia/reoxygenation, IL-1β, or a diabetic environment and compare CTHRC1 expression with fibrosis markers such as COL1A1, COL3A1, FN1, ACTA2, POSTN, VIM, and TGFB1. Such reanalysis will allow examination of the role of CTHRC1 and whether it is elevated solely as a marker in fibrotic organoids or whether it is associated with a specific fibroblast/myofibroblast subpopulation. There is also in silico evidence for a profibrotic role, but most of it comes from single-cell studies that have analyzed multiple organ fibrosis, not just the kidney. One study combined large-scale scRNA-seq data from patients with heart, lung, liver, and kidney fibrosis and demonstrated that fibroblasts and myofibroblasts are the main cells producing ECM [51]. In the same work, a myofibroblast subtype, designated CTHRC1_MyoFib, was described as one of the populations with the highest expression of collagen genes, supporting the association of CTHRC1 with fibrotic ECM-forming cells [51].

Three future directions are important to clarify the precise role of CTHRC1 in renal fibrosis: (1) reanalyze published kidney organoid RNA-seq/scRNA-seq data and compare CTHRC1 expression with fibrosis markers and cell clusters; (2) examine CTHRC1 in kidney organoid models induced by TGF-β1 or hypoxia/IL-1β by qPCR, immunostaining, and secreted protein/ELISA; (3) functionally evaluate its effects on ECM formation, myofibroblast activation, and tubular injury/regeneration by CTHRC1 knockdown or overexpression. Such studies will allow us to clarify whether CTHRC1 is simply a biomarker in renal fibrosis or a functional mediator that could be a therapeutic target.

### Future directions

In future studies, it is important to comprehensively determine the exact pathogenetic role of CTHRC1 protein in renal fibrosis. It is necessary to clarify how CTHRC1 expression changes in different cell populations of the kidney, including fibroblasts, pericytes, tubular epithelial cells, endothelial cells, and immune cells. To this end, the use of modern Omics technologies such as single-cell transcriptomics, spatial transcriptomics and proteomics will allow us to deeply understand the distribution of CTHRC1 in the foci of fibrosis and its activity at each stage of the disease.

The next important direction is to prove that CTHRC1 is not only a marker accompanying fibrosis, but also a factor directly contributing to the development of fibrosis. To this end, it is necessary to evaluate its effect on extracellular matrix formation, fibroblast activation, epithelial-mesenchymal transition, and inflammatory responses by inhibiting or increasing CTHRC1 expression in in vitro cell models, renal organoids, and animal fibrosis models. In addition, the study of the interaction of CTHRC1 with TGF-β, Wnt/PCP, Notch, Hippo and other profibrotic signaling pathways will help identify new molecular targets.

## Conclusion

CTHRC1 may be involved in fibrotic processes, tissue remodeling, and extracellular matrix turnover. From a clinical perspective, the potential use of CTHRC1 as an early biomarker of renal fibrosis is of particular interest. If its levels show a consistent relationship with fibrosis severity, renal function decline, and disease progression, CTHRC1 could be used to stratify patients according to risk and monitor treatment efficacy. Therapeutically, antifibrotic approaches targeting CTHRC1 may open the way for new treatment strategies in renal fibrosis and other tissue specific fibroses. However, their safety, efficacy, and renal selectivity must be thoroughly investigated before such approaches can be introduced into the clinic. Accumulating scientific evidence suggests that CTHRC1 may be a promising biomarker for tissue damage and specific functional states of fibroblasts. However, the specific role of CTHRC1 and its multifaceted activities require further investigation to better understand its s precise role as a direct enhancer of renal fibrosis, a feedback regulator limiting the fibrotic process, or a therapeutic target. The effects of CTHRC1 vary depending on cell type, tissue microenvironment, and disease stage, and may be contradictory in some cases. Therefore, elucidating the role of its context-dependent functions in the development and progression of kidney diseases is an important prerequisite for safe and effective therapeutic regulation of CTHRC1 activity. In this regard, the clinical relevance of CTHRC1 needs to be further confirmed in well-characterized patient cohorts, in functional models specific to kidney tissue, and by comprehensive analysis of kidney biopsy data.

## Data Availability

No datasets were generated or analysed during the current study.
